# Stromal Features of the Primary Tumor Are Not Prognostic in Genetically Engineered Mice of Pancreatic Cancer

**DOI:** 10.3390/cells9010058

**Published:** 2019-12-24

**Authors:** Marie C. Hasselluhn, Lukas Klein, Melanie S. Patzak, Sören M. Buchholz, Philipp Ströbel, Volker Ellenrieder, Patrick Maisonneuve, Albrecht Neesse

**Affiliations:** 1 Department of Gastroenterology and Gastrointestinal Oncology, University Medical Center, 37075 Göttingen, Germany; marie.hasselluhn@gmail.com (M.C.H.); lukas.klein@stud.uni-goettingen.de (L.K.); volker.ellenrieder@med.uni-goettingen.de (V.E.); 2 Institute of Pathology, University Medical Center, 37075 Göttingen, Germany; philipp.stroebel@med.uni-goettingen.de; 3 Division of Epidemiology and Biostatistics, IEO Istituto Europeo di Oncologia IRCCS, P.I. 08691440153 Milan, Italy; patrick.maisonneuve@ieo.it

**Keywords:** pancreatic cancer, tumor microenvironment, cancer-associated fibroblasts, activated stroma index, KPC model

## Abstract

The Kras^G12D/+^;LSL-Trp53^R172H/+^;Pdx-1-Cre (KPC) mouse model is frequently employed for preclinical therapeutic testing, in particular in regard to antistromal therapies. Here, we investigate the prognostic implications of histopathological features that may guide preclinical trial design. Pancreatic tumor tissue from n = 46 KPC mice was quantitatively analyzed using immunohistochemistry and co-immunofluorescence for proliferation (Ki67), mitotic rate (phospho-Histone 3, PHH3), apoptosis (cleaved caspase-3, CC3), collagen content, secreted protein acidic and rich in cysteine (SPARC), hyaluronic acid (HA), and α-smooth muscle actin (α-SMA). Furthermore, mean vessel density (MVD), mean lumen area (MLA), grading, activated stroma index (ASI), and fibroblast-proliferation rate (α-SMA/Ki67) were assessed. Univariate analysis using the Kaplan–Meier estimator and Cox regression model for continuous variables did not show association between survival and any of the analyzed parameters. Spearman correlation demonstrated that desmoplasia was inversely correlated with differentiated tumor grade (ρ = −0.84). Ki67 and PHH3 synergized as proliferation markers (ρ = 0.54), while SPARC expression was positively correlated with HA content (ρ = 0.37). MVD and MLA were correlated with each other (ρ = 0.31), while MLA positively correlated with CC3 (ρ = 0.45). Additionally, increased MVD was correlated with increased fibroblast proliferation rate (α-SMA + Ki67; ρ = 0.36). Our pilot study provides evidence that individual histopathological parameters of the primary tumor of KPC mice are not associated with survival, and may hint at the importance of systemic tumor-related effects such as cachexia.

## 1. Introduction

Pancreatic ductal adenocarcinoma (PDAC) is a highly lethal disease that is projected to become the second leading cause of cancer-related death in the United States and Germany by 2030 [1,2]. PDAC represents the predominant form of pancreatic cancer and is genetically characterized by oncogenic *KRAS* activation in 90% of all patients. Further genetic hallmarks of PDAC include frequent inactivation of tumor-suppressor genes such as *CDKN2A*, *DPC4*, and *TP53*. Importantly, PDAC is marked by a robust desmoplastic response with the stromal compartment comprising up to 90% of the bulk tumor [3]. For many years, the tumor stroma was understood to promote tumorigenesis and the spread of PDAC [4]. In particular, inflammatory signaling cues within the tumor microenvironment, activated stellate cells (PSCs), and cancer-associated fibroblasts (CAFs) were shown to promote tumor progression and metastatic disease [5,6,7,8,9]. Furthermore, abundant extracellular-matrix (ECM) components such as collagen or hyaluronic acid (HA) are deposited around neoplastic cells and increase the interstitial and intratumoral fluid pressure [10,11]. In contrast to other epithelial cancers, PDAC is characterized by pronounced hypovascularity, and tumor vessels are compressed by ECM components, further decreasing PDAC perfusion. Therefore, it was hypothesized that the pronounced desmoplastic reaction may impede drug accumulation and response in PDAC by creating biophysical barriers, thus mediating chemoresistance in PDAC [10,11,12,13].

Consequently, the tumor stroma has been perceived as an attractive therapeutic target, and multiple antistromal approaches have been tested over the last few years to break down the stroma barrier in order to increase drug delivery and sensitize tumors for chemotherapy [12,14,15,16,17]. Several approaches, such as inhibition of the sonic hedgehog signaling pathway, matrix metalloproteinases (MMPs), and depletion of HA by enzymatic inhibition initially showed promising preclinical or early clinical results, but failed at later stages of clinical testing [10,11,12,18]. As a result, there are currently no approved antistromal approaches for the clinical management of PDAC patients. More recently, in-depth investigations using genetically engineered mouse models (GEMMs) of PDAC have provided compelling evidence that the tumor stroma, and CAFs in particular, also mediate tumor-restraining properties, and that CAF depletion resulted in more aggressive, mesenchymal pancreatic tumors [19,20]. These conflicting data have caused considerable doubt about whether unselective depletion of cellular or acellular stromal components represents a beneficial therapeutic strategy.

Recent transcriptional and functional data show tremendous heterogeneity and complexity of the tumor microenvironment in PDAC, including several subtypes of tumor-promoting, tumor-restraining, or antigen-presenting CAFs [7,8,9,21,22], and a highly heterogeneous distribution and content of ECM components in PDAC [23]. To this end, transcriptional data have identified various stromal subtypes that correlate with patient survival [24,25]. Depending on the datasets, an ‘immune-rich’ subtype was characterized by numerous immune and interleukin signals, whereas the ‘ECM-rich’ subtype showed abundant ECM-associated pathways [24].

Currently, the Kras^G12D/+^;LSL-Trp53^R172H/+^;Pdx-1-Cre (KPC) mouse model remains the most frequently used model system in PDAC research due to its heterogeneity, stromal response, and close recapitulation of clinical features of human PDAC such as ascites, jaundice, liver metastases, and cachexia [26]. However, it is still unclear whether histological features, in particular, stromal determinants such as the activated stroma index (ASI), are prognostic in KPC mice [27]. Alternatively, the occurrence of ascites, bile-duct obstruction, or cachexia may be critical for survival, independently from histological features of the tumor. This is of particular interest in light of the discrepancy between successful preclinical stromal therapies and subsequent failure in clinical trials in regard to the antitumoral response of drugs and survival analysis.

Here, we aim to characterize key stromal and epithelial components in KPC mice by immunohistochemistry to identify prognostic markers that may guide preclinical trial design and interpretation.

## 2. Materials and Methods

### 2.1. Mouse Strains

Kras^G12D^, Tp53^R172H^, and Pdx-Cre mouse strains were interbred to obtain KPC mice [26]. Successful generation of triple mutant animals was confirmed by genotyping PCR. The following end-point criteria were employed: development of ascites, body-weight loss >20%, decline of body temperature, piloerection, lethargy, and reduced social behavior. All animal breeding was carried out using protocols approved by the Institutional Animal Care and Use Committee at the University Medical Center Göttingen. Mice were housed in a 12 h light, 12 h dark rhythm.

### 2.2. Tumor Evaluation

Histological confirmation of established invasive PDAC in all mice included in this study was performed by the Institute of Pathology, University Medical Center, Göttingen. Specifically, murine PDAC was graded in terms of tumor differentiation (G1–3) and desmoplasia (D1–2). Although different desmoplasia and differentiation grades were observed in 1 tumor, the predominant phenotype was used for final grading.

### 2.3. Immunohistochemistry (IHC) and Immunofluorescence (IF) Staining

KPC mice were sacrificed upon achieving one of the aforementioned end-point criteria, and tissue samples were fixed in 10% neutral-buffered formalin (Sigma, St. Louis, MO, USA) for 24 h before transferring to 70% ethanol and subsequently to paraffin embedding. Hematoxylin and eosin (H and E), IHC, and IF were performed on 3–5 µm sections using standard protocols as previously described [14,28]. Picrosirius Red staining (Polysciences, Inc.; 24901; Warrington, PA, USA) was conducted according to the manufacturer’s instructions. Utilized antibodies are listed in Table 1.

CC3, Ki67, PHH3, and CD31 stainings were quantified by an Olympus BX51 microscope combined with a Leica Aperio XT automated scanning system and subsequent automated analysis with Aperio Imagescope 10 software. For each KPC mouse, 30 pictures per staining were analyzed, and adjacent PanINs, ADMs, and necrotic areas were left out of analysis. Mean vessel density (MVD) and mean lumen area (MLA) were extracted from CD31 staining and calculated by Imagescope 10.

To assess α-SMA and Ki67 expression via IF, 5 pictures per mouse (400× magnification) were quantified by measuring the amount of all nuclei surrounded by positive α-SMA signal and subsequent counting of double-positive (α-SMA + Ki67) nuclei. The ratio was evaluated for each picture, respectively, and values were averaged per mouse. Area staining for Picrosirius Red, hyaluronan-binding protein (Habp), α-SMA, and SPARC were analyzed by planimetrical analysis using Fiji (WI, USA) [29]. Picture acquisition was realized under strict compliance with regards to exposure time, light intensity, contrast, aperture, and sensor sensitivity (ISO). Macros were designed to enable uniform signal detection of different areas and among all mice by defining thresholds for hue, saturation, and brightness.

### 2.4. Statistical Analysis

The primary study end-point was survival, calculated from date of birth to date of death of the mouse. Univariable analysis was based on Kaplan–Meier plots separating strong and weak signals of respective staining by the median and a univariate Cox proportional hazard regression model. Quantile-Quantile Plot by Statistica (version 13.0; Tulsa, OK, USA) confirmed the Gaussian distribution of measured variables. Graph plotting and statistical analysis by the log-rank (Mantel–Cox) test were performed by GraphPad Prism (version 8.3.0; San Diego, CA, USA).

Additionally, measured parameters were integrated into multivariable regression analysis by Spearman Rank Order Correlations to identify the association of parameters. Statistical analysis was conducted in Statistica (version 13.0; Tulsa, OK, USA).

## 3. Results

### 3.1. Association of Tumor Grading and Survival Not Confirmed

**Figure 1:**
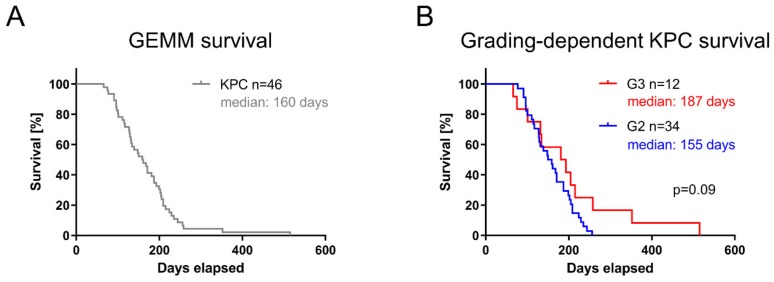
Pancreatic ductal adenocarcinoma (PDAC) heterogeneity in Kras^G12D/+^;LSL-Trp53^R172H/+^;Pdx-1-Cre (KPC) mouse model. (**A**) Survival of KPC cohort (n = 46) with median survival of 160 days. (**B**) PDAC establishment histopathologically confirmed and respective tumors graded.

Apart from an activating *Kras^G12D^* mutation, the KPC model relies on an additional inactivating point mutation in tumor suppressor gene *Tp53*, thus leading to a dramatic acceleration of PDAC in KPC mice (median survival: 160 days, n = 46; Figure 1A). Tumor grading in KPC mice confirmed heterogeneity in differentiation, as observed by others [26] (Figure 1B, Supplementary Figure S1). Tumor grading did not reveal an association with animal survival (median survival 155 days versus 187 days, log-rank *p* = 0.09; Figure 1B). Importantly, diverse grading of KPC-derived tumors resembles PDAC heterogeneity as observed in patients [26,30]. Together, these results demonstrated no prognostic value of tumor grading based on overall survival in the KPC cohort.

### 3.2. Assessment of Proliferation and Apoptosis Rate Is Insufficient in Predicting KPC Mice Survival

**Figure 2:**
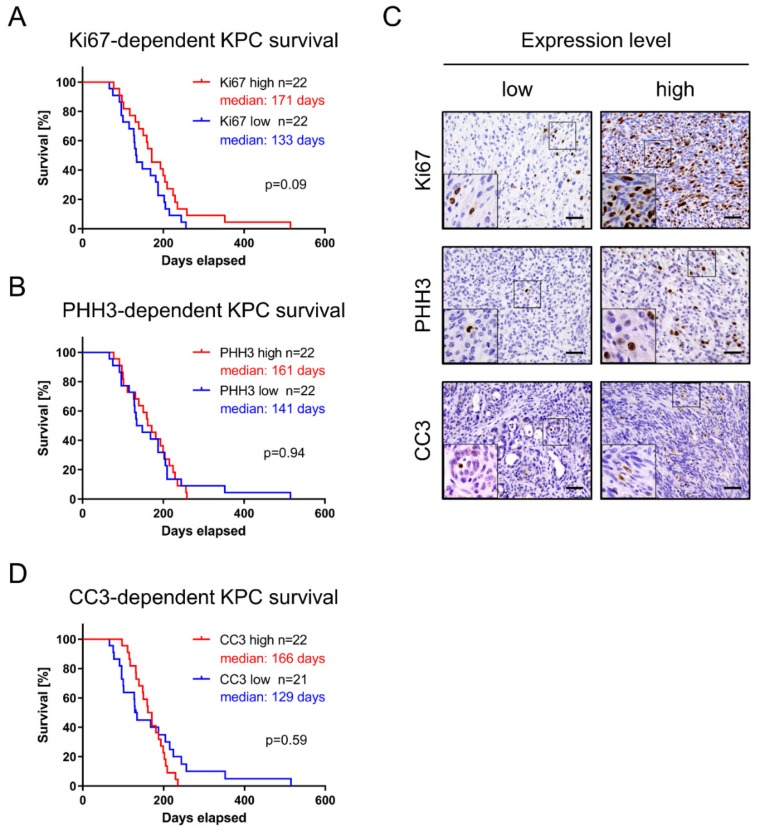
Proliferation and apoptosis markers in KPC mice. Proliferation was investigated via (**A**) Ki67 and (**B**) PHH3. (**C**) IHC staining and subsequent quantification enabled differentiation between high- and low-expressing tumor tissue, respectively. Scale equals 50 µm. (**D**) Apoptotic rate assessed via CC3 expression.

Malignant tumors are characterized by invasiveness, dedifferentiation, uncontrolled cellular growth, and apoptosis evasion. Therefore, we assessed a number of key histopathological features by IHC in KPC mice and systematically quantified these parameters (Table 2). The discriminator between low and high expression was set as the median of the respective value in the cohort (Table 2). Expression of proliferation markers Ki67 (median survival 171 days versus 133 days; log-rank *p* = 0.09) and PHH3 (median survival 161 days versus 141 days; log-rank *p* = 0.94) disclosed no significant association to overall survival (Figure 2A–C).

In addition, we tested apoptosis marker CC3 in KPC-derived PDAC, and found no association between high- and low-expressing cohorts and median survival (median survival 166 days versus 129 days; log-rank *p* = 0.59; Figure 2C,D). We thus conclude that association of survival with common diagnostic markers such as Ki67, PHH3 or CC3 could not be confirmed in KPC mice.

### 3.3. Fibroblast Infiltration of PDAC Is Not of Prognostic Value

**Figure 3:**
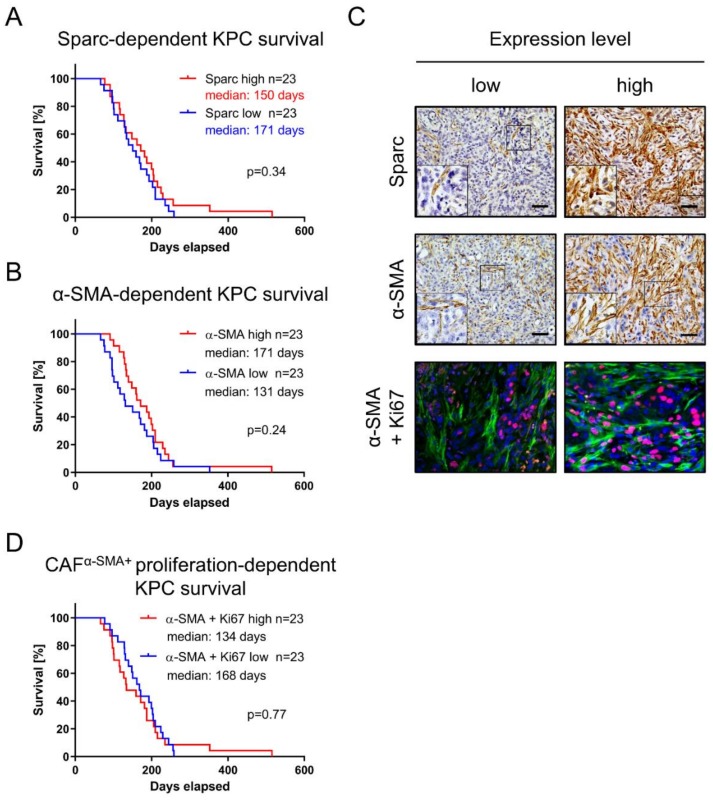
Fibroblast-dependent KPC survival. Fibroblast-specific markers (**A**) Sparc and (**B**) α-SMA were examined for their prognostic value in KPC model. (**C**) Quantification based on IHC or IF, respectively, dividing KPC cohort into low- and high-expressing tumors. Scale equals 50 µm. (**D**) cancer-associated-fibroblast (CAF) proliferation assessed via IF, quantifying proportion of Ki67 and α-SMA double-positive cells in murine PDAC tissue.

PDAC is markedly shaped by infiltrating CAFs contributing to the dominant stromal compartment [3]. Both SPARC and α-SMA are CAF markers. Interestingly, while SPARC was found to be highly upregulated in the “activated” stroma subtype associated with poor prognosis [25], α-SMA was described as a marker for tumor-restraining myofibroblastic CAFs (myCAFs) [8]. Accordingly, we investigated the potential association of both CAF markers for survival in KPC mice. The Sparc-high cohort revealed a median survival of 150 days, whereas the Sparc-low cohort showed 171 days median survival (log-rank *p* = 0.34; Figure 3A,C). Accordingly, no association was confirmed for α-SMA expression and survival in KPC mice (median survival 171 days versus 131 days; log-rank *p* = 0.24; Figure 3B,C). We further assessed the implication of proliferating CAFs by costaining of Ki67 and α-SMA using IF (Figure 3C,D), but failed to show significant impact on KPC mouse survival (median survival 134 days versus 168 days; log-rank *p* = 0.77).

### 3.4. Quantity of Collagen and Hyaluronic Acid Is Not Associated with KPC Survival

**Figure 4:**
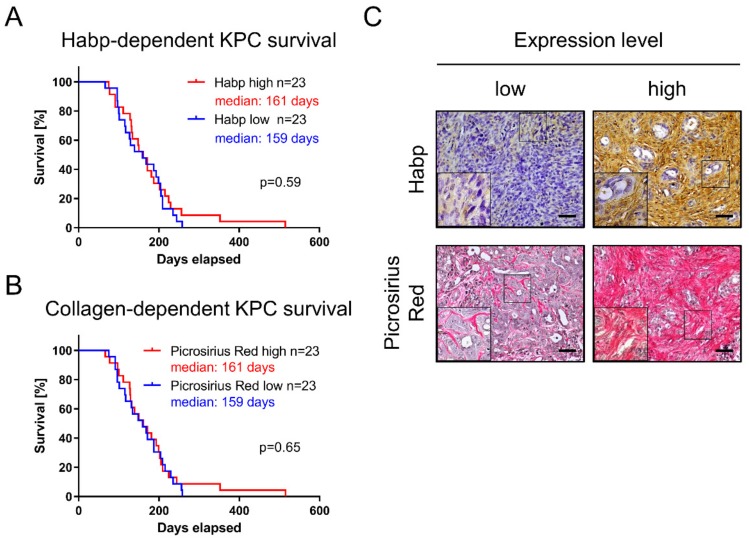
Influence of tumor-stroma composition on KPC survival. Extracellular-matrix (ECM) components (**A**) Habp representing hyaluronic acid (HA) and (**B**) collagen via Picrosirius staining were investigated with regard to KPC survival. (**C**) Representative pictures for low and high expression levels, respectively. Scales equal 50 µm.

PDAC heterogeneity does not only cover the mutational landscape of epithelial tumor cells, but also affects the tumor microenvironment and ECM composition [30]. Major ECM proteins of the PDAC microenvironment include collagen and HA. Therefore, we stained KPC tumors for Habp and collagen (Picrosirius Red) to investigate their correlation with overall survival. However, the association between HA (median survival 161 days versus 159 days; log-rank *p* = 0.59) and collagen content (median survival 161 days versus 159 days; log-rank *p* = 0.65) was rejected (Figure 4A–C).

### 3.5. Increased Vascularization Does Not Augment KPC Survival

**Figure 5:**
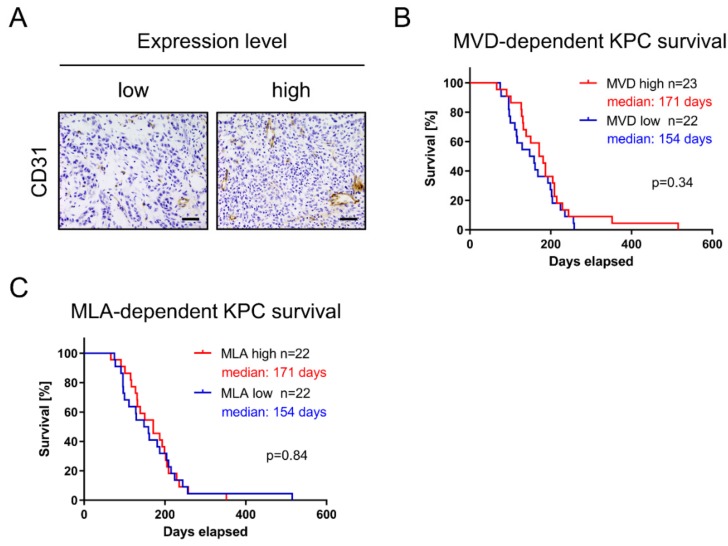
KPC survival not dependent on tumor vascularity. (**A**) IHC for CD31 performed to evaluate tumor vascularization. Scale equals 50 µm. Vascularity and compression of tumor vessels were investigated by scrutinizing (**B**) MVD and (**C**) MLA.

The PDAC tumor microenvironment is characterized by a paucity of vessels. It has been hypothesized that this hypovascularity contributes to therapeutic resistance by impeding the drugs’ ability to penetrate the tumor [10,11,31,32]. However, it is unclear whether vascularization or perfusion of tumors in KPC mice has prognostic implications. Therefore, we assessed tumor vascularity by CD31 IHC (Figure 5A). Immunohistochemical staining was automatically quantified for MVD and MLA as a surrogate for tumor perfusion. No survival association between the MVD-high and MVD-low cohorts could be confirmed (median survival 171 days versus 154 days; log-rank *p* = 0.34; Figure 5B). In addition, the association between MLA-high and MLA-low cohorts was rejected (median survival 171 days versus 154 days; log-rank *p* = 0.84; Figure 5C).

### 3.6. Extent of Desmoplasia and Activation Status of Stroma Do Not Influence KPC Survival

**Figure 6:**
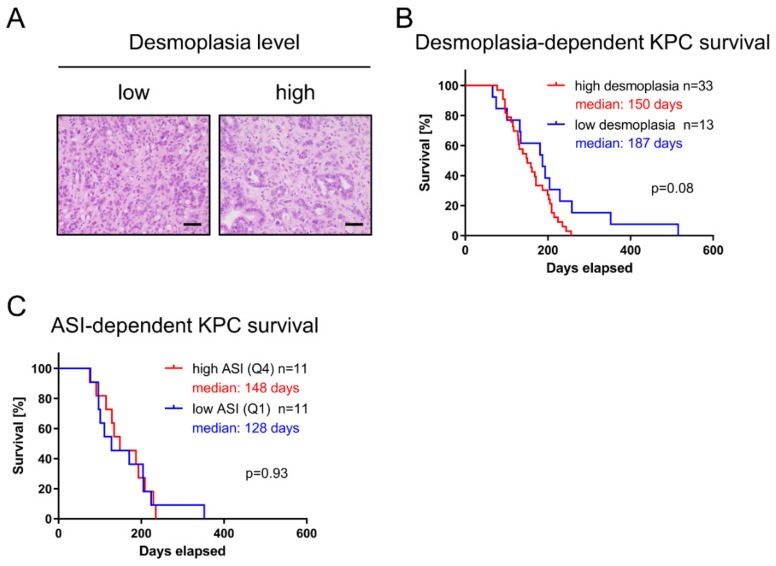
Desmoplasia and fibroblast activation not correlated to KPC survival. (**A**) Hematoxylin and eosin (H&E) staining revealed heterogeneity in desmoplasia despite equal grading (G2). (**B**) H&E staining graded according to extent of tumor stroma. (**C**) Activated stroma index (ASI) calculated from the ratio of α-SMA positive area and collagen positive area and subdivided into quartiles.

Desmoplasia describes excessive fibrosis marked by the accumulation of ECM proteins and fibroinflammatory cells that surround neoplastic cells. Therefore, KPC tissue was semiquantitatively scored by an expert pathologist (D1 = low desmoplasia; D2 = high desmoplasia), as illustrated in Figure 6A. No association between murine pancreatic tumors with high or low amounts of tumor stroma and survival could be established (median survival 150 days versus 187 days; log-rank *p* = 0.08; Figure 6B). On the basis of the ratio of the α-SMA positive area and collagen positive area (Picrosirius Red), the ASI was assessed; resulting ratios were subdivided into quartiles, and the lowest and highest quartiles were compared as previously described by Erkan et al. [27]. In contrast to data obtained from patient-derived PDAC tissue [27], the ASI had no prognostic value in KPC mice (median survival 148 days versus 128 days; log-rank *p* = 0.93; Figure 6C).

### 3.7. Univariate Analysis for Continuous Variables Using Cox Regression Model

To test whether very high or very low parameter values were correlated with the survival of KPC mice, we performed univariate analysis for continuous variables using the Cox regression model. However, none of the analyzed parameters was significantly associated with the survival of KPC mice. Results are summarized in Table 2. In addition, Spearman Rank Order Correlation was utilized to identify six pairs of tested parameters with significant correlation (*p* < 0.05) (Supplementary Table S1). Importantly, a high degree of desmoplasia was inversely correlated with differentiated tumor grade (ρ = −0.84). Ki67 and PHH3 were positively correlated as proliferation markers (ρ = 0.54), while Sparc expression was positively correlated with Habp detection (ρ = 0.37). MVD and MLA synergized with each other (ρ = 0.31), while MLA was positively correlated with CC3 (ρ = 0.45). Additionally, increased MVD was correlated with increased fibroblast proliferation rate (α-SMA + Ki67; ρ = 0.36).

## 4. Discussion

Since its introduction in 2005, the KPC mouse model has revolutionized pancreatic-cancer research, in particular in terms of preclinical therapeutics and testing novel antistromal therapies [26,33]. Histological hallmarks are a pronounced tumor microenvironment with hypovascularity, hypoxia, CAF activation, ECM deposition, and immune cell infiltration. In addition, clinical presentation with malignant ascites, cachexia, metastasis formation, and biliary obstruction seem to recapitulate features of the human disease [26]. However, the KPC model relies on the early embryonic activation of *Pdx-Cre* recombinase, driving the expression of mutant *Kras^G12D^* and *Tp53^R173H^*, and resulting in early multifocal lesion formation throughout the whole pancreas. In addition, the *Pdx* promoter is not exclusively expressed in the pancreas and could subsequently cause papillomas of the skin, esophagus, and vulva, hampering preclinical trial enrollment.

Despite the wide use of the KPC mouse model, the translation of preclinical findings in KPC mice to clinically successful compounds has been challenging. So far, the pharmacological depletion of HA by enzymatic degradation using PEGPH20 in combination with gemcitabine was the most promising finding in GEMMs [10,11], and the corresponding phase I/II trial [34]. However, the currently unpublished randomized phase III trial (NCT02715804) using PEGPH20 in combination with nab-paclitaxel and gemcitabine in stage IV PDAC patients with high levels of HA was recently halted and is expected to not meet its end points. Therefore, it is currently not clear whether GEMMs truly have more predictive power than that of other models, such as orthotopic or subcutaneous tumor models. To this end, we sought to systematically investigate the histopathological features of KPC tumors for possible associations with survival that may help to guide and control preclinical trial design, and respond to experimental antineoplastic agents. To the best of our knowledge, such a systematic assessment has not been performed before in KPC tumor tissue.

In our study, we employed KPC mice on the basis of their tumor heterogeneity, and investigated histopathological parameters that were differentiated by median or continuous variables. Our results, using both Kaplan–Meier plots and a univariate Cox regression model, clearly showed that single parameters are not correlated with KPC survival. Thus, our results highlighted the distinct differences between KPC mouse histology and human PDAC. For instance, the amount of HA was shown to be correlated with worse survival in PDAC patients [35,36]. Furthermore, stromal SPARC expression was correlated with poor prognosis in PDAC patients [37]. In addition, the ASI was identified in resected PDAC patients and was correlated with worse survival, indicating a CAF tumor-promoting effect that we could not recapitulate in KPC mice [27].

Future approaches should investigate the proliferative capacity of several CAF subpopulations, such as SAA3, FAP-α, and PDGFR-β, expressing CAFs before and after treatment [9,38]. Interestingly, fibroblast proliferation was positively correlated with MVD, indicating a potential role of CAFs in regulating tumor vascularity. Furthermore, overall desmoplasia demonstrated strong negative correlation with tumor grading, suggesting a potentially protective role of the tumor stroma on PDAC differentiation status. However, the ASI could not be shown to have a predictive value in KPC mice, which is in contrast to results from human PDAC [27], and may point towards distinct differences between human and mouse stroma composition.

Our preclinical study has several limitations. First, we presented a small and descriptive pilot study with a limited number of KPC mice. Second, our analysis did not allow us to draw causative conclusions on tumor biology, but rather provided markers that should be validated in future preclinical trials using KPC mice. Third, multivariable analysis would have complemented this type of study, which we performed, but the obtained results were not valid in this cohort of mice due to multicollinearity.

In conclusion, our study analyzed a set of histopathological parameters by IHC and co-IF in a cohort of untreated KPC mice. Our results using univariable analysis suggested that single histopathological parameters are not prognostic for KPC survival. This is in contrast to data in human PDAC, where markers such as HA, ASI, and SPARC have been shown to be correlated with patient outcome. Therefore, great care should be taken with the interpretation of preclinical results in KPC mice. Not only histological parameters of the primary tumor, but also systemic tumor-related symptoms such as ascites, jaundice, and cachexia should be recorded and systematically evaluated (e.g., by muscle biopsies) in response to treatment.

## Figures and Tables

**Table 1:** Antibodies used for immunohistochemistry (IHC) and immunofluorescence (IF) staining. Abbreviations: α-SMA, α-smooth muscle actin; SPARC, secreted protein acidic and rich in cysteine.

Antibody	Company	No.	Application
CC3	Cell Signaling Technology, Inc.; Danvers, MA, USA	9661/9664L	IHC
CD31	BD Pharmingen; Franklin Lakes, NJ, USA	553370	IHC
Habp	Merck Millipore; Billerica, MA, USA	385911	IHC
PHH3	Upstate/Merck Millipore; Billerica, MA, USA	06-570	IHC
Ki67	Thermo Fisher Scientific, Inc.; Waltham, MA, USA	RM-9106-S0	IHC/IF
α-SMA	Dako Denmark A/S; Glostrup, Denmark	M0851	IHC/IF
SPARC	R and D Systems, Inc.; Minneapolis, MN, USA	AF942	IHC

**Table 2:** Results from univariable histopathological analysis in KPC tumor tissue for all analyzed parameters. Significance of association with survival was calculated by univariate Cox regression model. Abbreviations: SD, standard deviation; CI, confidence interval; IQR, interquartile range; HR, hazard ratio; MVD, mean vessel density; MLA, mean lumen area; * for continuous variables, HR and 95% CI presented for increase corresponding to IQR.

	Number	Mean ± SD	Median Q1–Q4)	HR (95% CI)	*p*-Value
***Ki67***	44	10.6 ± 4.8	9.41 (7.05–12.9)	0.68 (0.43–1.05) *	0.08
***CC3***	43	0.80 ± 0.98	0.51 (0.32–0.99)	0.97 (0.78–1.21) *	0.79
***PHH3***	45	0.41 ± 0.27	0.37 (0.22–0.46)	1.09 (0.82–1.45) *	0.53
***Picrosirius***	46	22.1 ± 9.8	21.9 (14.3–29.0)	0.93 (0.59–1.48) *	0.77
***Habp***	46	13.2 ± 8.5	11.2 (7.72–16.3)	0.96 (0.70–1.31) *	0.79
***Sparc***	46	5.1 ± 2.2	4.63 (3.48–6.18)	0.89 (0.59–1.36) *	0.61
***α–SMA***	46	3.4 ± 2.8	2.81 (1.72–4.49)	0.90 (0.63–1.28) *	0.56
***α–SMA+Ki67***	46	6.7 ± 3.4	6.27 (4.25–7.79)	1.27 (0.90–1.79) *	0.17
***MLA (CD31)***	45	37.1 ± 11.0	34.6 (30.5–46.4)	0.75 (0.45–1.25) *	0.27
***MVD (CD31)***	45	173 ± 64	167 (121–219)	0.98 (0.58–1.66) *	0.79
***Grading (G1 vs. G3)***	46	-	-	0.53 (0.25–1.13)	0.10
***Desmoplasia (D1 vs. D2)***	46	-	-	1.88 (0.91–3.87)	0.09
***ASI***	46	0.23 ± 0.35	0.11 (0.06–0.24)	0.95 (0.82–1.10) *	0.47

## Supplementary Materials

The following are available online at https://www.mdpi.com/2073-4409/9/1/58/s1. Table S1: Spearman Rank Order Correlation (ρ). Figure S1: H and E staining for different histological variants in KPC tumors.
